# Country score tool to assess readiness and guide evidence generation of immunization programs in aging adults in Europe

**DOI:** 10.3389/fpubh.2022.1080678

**Published:** 2023-01-09

**Authors:** Thi Hao Pham, Ekkehard Beck, Maarten J. Postma, Bertalan Németh, Tamás Ágh, Chiara de Waure, David M. Salisbury, Nynke Nutma, Jurjen van der Schans

**Affiliations:** ^1^Unit of Global Health, Department of Health Sciences, University Medical Center Groningen, Groningen, Netherlands; ^2^Asc Academics, Groningen, Netherlands; ^3^Department of Vaccines Value Evidence, GlaxoSmithKline, Wavre, Belgium; ^4^Department of Economics, Econometrics & Finance, University of Groningen, Groningen, Netherlands; ^5^Centre of Excellence in Higher Education for Pharmaceutical Care Innovation, Universitas Padjadjaran, Bandung, Indonesia; ^6^Syreon Research Institute, Budapest, Hungary; ^7^Department of Medicine and Surgery, University of Perugia, Perugia, Italy; ^8^Royal Institute International Affairs, Chatham House, London, United Kingdom; ^9^RIVM, The Dutch National Institute for Public Health and the Environment, Bilthoven, Netherlands

**Keywords:** immunization programs, aging, readiness, assessment, evidence, tool

## Abstract

**Objectives:**

Delaying of policies for immunization of aging adults, low vaccine uptake, and the lack of supportive evidence at the national level could diminish the value in health and economics of such programs. This study aims to develop a “country score tool” to assess readiness and to facilitate evidence generation for aging adult immunization programs in Europe, and examine the comprehensiveness, relevance, acceptability, and feasibility of the tool.

**Methods:**

The tool was developed in two phases. First, a modified Delphi process was used to construct the tool. The process included a literature review, stakeholder consultations, and a three-round Delphi study. The Delphi panel included researchers, supra-national and national decision-makers of immunization programs recruited from five countries, using snowball sampling method. The consensus was predefined at the agreement rate of 70%. Pilot testing of the tool was conducted in the Netherlands, Germany, Serbia, and Hungary involving researchers in the field of health technology assessment. After assessing the countries' readiness, researchers evaluated four features, namely comprehensiveness, relevance, acceptability, and feasibility of the tool *via* an online survey that included 5-scale Likert questions. The percentages of affirmative answers including “agree” and “totally agree” choices were presented.

**Results:**

The review identified 16 tools and frameworks that formed the first version of our tool with 14 items. Eight experts were involved in the Delphi panel. Through three Delphi rounds, four items were added, one was dropped, and all others were amended. The consensus was achieved on the tool with 17 items divided into decision-making and implementation parts. Each item has a guiding question, corresponding to explanations and rationales to inform assessment with readiness scores. Eight researchers completed the pilot testing. The tool was rated as comprehensive (75%), relevant (100%), acceptable (75%), and feasible (88%) by participants.

**Conclusion:**

Through a thorough and transparent process, a country score tool was developed helping to identify strengths, weaknesses, and evidential requirements for decision-making and implementation of immunization programs of aging adults. The tool is relevant for different European contexts and shows good comprehensiveness, acceptability, and feasibility.

## 1. Introduction

Burden of vaccine preventable diseases (VPDs) is threatening healthcare systems not only by an increasing number of aging people (50 years and older) but also through the age-related decline of the immune system (1, 2). An extreme burden was demonstrated with the COVID-19 pandemic when a sudden increase in cases, morbidity and mortality became an urgent problem (3). Besides COVID-19, other VPDs significantly impact on aging population. The issue is exemplified by European data with ~40,000 deaths annually caused by influenza with 90% occurring among elderly (4), 73 hospitalizations per 100,000 seniors every year for community-acquired pneumococcal pneumonia (5), and almost a third of adults developing herpes zoster over lifetime (6).

As a preventive strategy, vaccination has been reported to be effective in limiting the severity, reducing morbidity and mortality of infections in aging population (7–9). Those health benefits enable them to contribute to nations' social and economic development as well as perform various valuable functions including childcare, providing financial and emotional support to families (10). Along with health and social benefits, immunization programs have been proved to be cost-effective (11–13). Despite those potential benefits in health, social and economic aspects, vaccines are underutilized in aging population characterized by delayed policy implementation and low vaccine uptakes. Less than two third of European countries had policies for pneumococcal vaccine and most countries had no vaccination policies against herpes zoster (14). Although influenza vaccination programs were in place all over Europe, only one country achieved the vaccine uptake target in 2018/2019 of 75% recommended by World Health Organization (WHO) (15, 16). During the same season, half of the countries had <35% of older people vaccinated against influenza; the lowest coverage was <1% (16). Therefore, countries need to strengthen their immunization programs for aging population by focusing on both decision-making and implementation aspects.

Required elements for both decision-making and strategies for implementation have been investigated among European countries. The most important component for making decision on vaccine introduction is evidence, including international literature and country-specific evidence (17). The former can be done by a standardized systematic approach using available supporting tools, for instance, GRADE and CAPACITI (18, 19). The latter element includes, but is not limited to, setting-specific evidence of the burden of disease and health economic evaluation. However, in many countries, there is no transparent guidance that presents various methodological requirements (17). It suggests a need for a holistic assessment to first identify the gaps in healthcare systems concerning aging adult immunization programs, and second, to identify methods for future research enabling decision-making and implementation (20). This assessment could be sufficiently defined by a concept of a “readiness assessment.” By definition, readiness assessment can help to identify the potential challenges when implementing new processes within a current organizational context and affords the opportunity to remedy these gaps either before, or as part of, the implementation plan (21).

Although the two concepts of “decision-making” and “implementation” are commonly used across publications (22–24), they are poorly defined. That could be driven from the complexity and dynamic nature of introducing vaccines into different settings. A recent study identified key features of vaccine market access pathways across the European Union (EU) and the United Kingdom. They included horizon scanning, early advice, initiation of assessment, recommendations from advisory groups for vaccine introduction and funding, final decision, National Immunization Program (NIP) inclusion, and procurement (25). Drawing on that pathway, we defined decision-making as the process of generating recommendations to include vaccines to the national immunization program and possibly reimbursed by National Immunization Technical Advisory Group (NITAG) and/or Health Technology Assessment Body (HTAB) (or equivalents); and implementation as the process of proceeding with the recommendations including resourcing, making final decisions of vaccine introduction, procurement, and sustainability.

Various published works exist that help to assess a country's readiness with regards to immunization programs; however, there are limitations in terms of target diseases, population, and transferability in different European contexts. First are readiness assessment tools for COVID-19 vaccine introduction, which might be though inapplicable for non-pandemic vaccines, given the special priority of resources for COVID-19 (26, 27). Second, a majority of guidelines and publications focus on vaccination of the general populations (28, 29), or specifically for children (30) or during pregnancy (31). Despite immunization programmes' common aspects, those of aging population have specific requirements including a need for adult vaccine working groups (22), evidence on potential gains from immunization, and relevant infrastructure to deliver immunization service to this population (20). Therefore, it is important to consider those existing guidelines as a foundation, but unique requirements for aging adults should be added to the assessment tool targeting this population. To partly fill in this gap, an “Evidence-Based Tool for Planning and Evaluating” was considered to be the first proposal up to 2021 aiming at improving coverage for the elderly by planning and monitoring vaccination strategies (32). However, the ability to support making policies and possibilities to generate evidence in immunization programs are not in scope of the tool. Additionally, the transferability of the tool to different European countries is limited when its development solely considered the Italian context.

All those gaps in the literature raise a need for a tool that can simultaneously assess countries' readiness and facilitate evidence generation to support decision-making and implementation processes of immunization programs in European countries targeting aging population. This work aims to (1) develop a country score tool, which can help to achieve those purposes and (2) to test the comprehensiveness, relevance, acceptability, and feasibility of the tool.

## 2. Materials and methods

**Figure 1:**
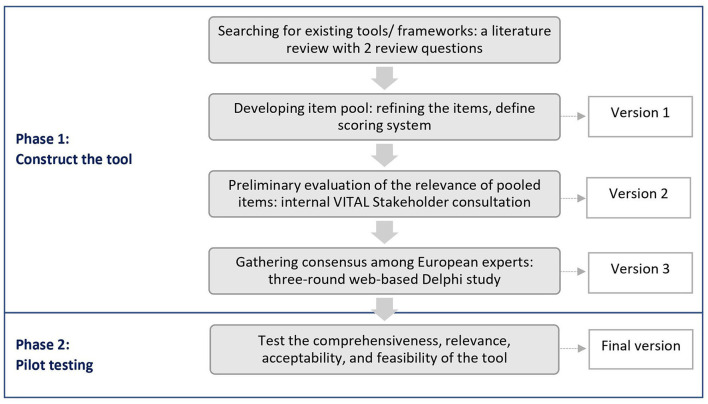
Development process of the country score tool.

The country score tool was developed in two phases (Figure 1). In phase 1, a modified Delphi process was used. This method concerns a group consensus strategy that systematically uses literature review, opinions of the research team (labeled steering group) and the judgment of experts within the research field to reach agreement (33, 34). Phase 2 involved pilot testing in four countries including the Netherlands, Germany, Hungary, and Serbia.

### 2.1. Phase 1: Tool development using a modified Delphi process

#### 2.1.1. Development of an item pool and the first version of the tool

We conducted a comprehensive literature review in August 2021 to identify existing readiness assessment tools and frameworks for immunization programs with regards to decision-making and implementation in general populations and adult populations separately. Publications that specifically focused on aging population were separated from the adult-related search. To define aging population, the cut-off of 50-year-old and over was applied as suggested in published work regarding vaccination to promote healthy aging globally (35, 36). Details of the literature review including inclusion, exclusion criteria, and search strategy are presented in Supplementary material 1. All components and sub-components of the identified tools and frameworks were extracted for the development of an item pool. COVID-19 related aspects were excluded in the development process. This feature enables the tool to be used for non-pandemic vaccination programs in aging population in the future after the COVID-19 pandemic era.

We structured the tool along the two lines of decision-making and implementation. The foundation for item generation was set up based on (1) a published systematic review of 116 studies on national decision-making for the introduction of new vaccines and (2) WHO guidance on principles and considerations for adding a vaccine to a national immunization program (28, 30). Next, items in the item pool were linked to this foundation. Similar components were grouped, and new components were added. Disease-specific or vaccine-specific items were excluded from the item bank afterwards.

The structure of the tool was generated by integrating a checklist approach and a scoring approach. As a checklist, the country readiness tool presented each item in the item pool as a question supported by definitions and rationales (31). In addition, the readiness level in each item was defined by a scoring system of three values including 0, 1, and 2 (22). No weighting system was implied in order to provide flexibility for countries when using the tool according to particular infrastructures and the national priorities.

#### 2.1.2. Stakeholder consultation

Consultation with stakeholders occurred in December 2021-January 2022 to first determine the relevance and importance of a set of components identified in the previous step, and to identify additional components. Participants were recruited from VITAL consortium comprising 17 public partners from 11 countries in Europe together with seven biopharmaceutical companies in a European project named Vaccines and InfecTious diseases in the Aging population (37).

All participants were provided with the first version of tool and asked for individual feedback before participating in an online meeting where all disagreements among individuals and suggestions for additional items were discussed. Polls were used during the meeting to support reaching agreements. The meeting was recorded and later transcribed *via* Microsoft Teams. The second version of the tool was generated according to the agreement.

#### 2.1.3. Three-round Delphi study

Delphi panelists were non-VITAL members who have experience in one or more of the predefined fields/positions: (1) Ministry of Health; (2) NITAG (or equivalent); (3) Insurer; (4) HTAB (or equivalent); (5) Representatives of: Healthy aging, General practitioners (GPs), Pharmacists, Nursing homes; (6) World Health Organization; and/or (7) Researcher in related fields including, but not limited to, Infectious diseases, Immunization, Epidemiology, Health Economics and Public Health. Snowball sampling method was used to recruit participants. This method involves identifying index individuals and asking them to refer other persons suitable for the study (38). Responses were collected *via* a Qualtrics survey using Qualtrics software (39).

**Figure 2:**
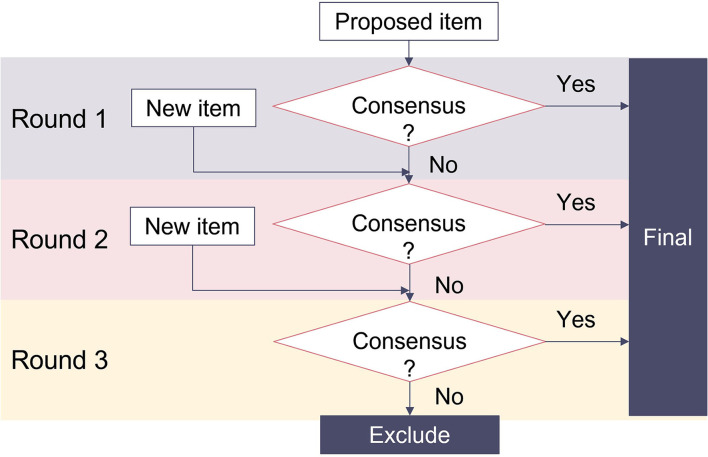
Item generation process through a three-round Delphi study.

Figure 2 presents the item evaluation process following the three-round Delphi study. In the first and second rounds, panelists were required to evaluate all items and give their opinions if the item should be “included as is,” “included with edits” or “dropped” from the tool; provide their suggestions for amendments, or for additional items or reasons for the exclusion. Consensus was predefined as a rate of agreement or disagreement above 70%. Items that reached the consensus value were included directly in the final version without further evaluation. Other items that did not reach the consensus or were subjects for edits or were newly added were transferred to the next round. Within 1 week after the evaluation period, panelists received the result and the survey link for the next round. Panelists who did not participate in one round were allowed to join the following round after he or she agreed with the current report.

In the third round, panelists were required to evaluate revised items resulting from round 2 by choosing “include the item” or “drop the item.” Suggestions for edits or new items were not applicable. Consensus for inclusion was defined with a cut-off of 70%. The final result of the Delphi study was made available for all participants 2 weeks after the third round.

### 2.2. Phase 2: Pilot testing

An e-pilot test was conducted in four European countries: Netherlands (NL), Germany (GER), Serbia (SER), and Hungary (HU) to (i) test comprehensiveness, relevance, acceptability, and feasibility of the tool (40); (ii) finalize the tool; and (iii) present preliminary assessment of the countries' readiness. Tool version 3 (electronic version in excel) was tested in a small sample of the target audience—two researchers per country in the field of health economics/ health policy. These criteria were made based on considering the feasibility of the project and the ability of participant to assess different aspects of the tool rather than the importance of their roles in the topic area. Having a good understanding of various dimensions in the vaccine introduction process, starting from measuring the burden of diseases to financing and delivering the intervention, health economic/ health policy participants are a relevant proxy of the multidisciplinary audience of the tool. Snowball sampling method was used to recruit participants.

First, participants were asked to complete the readiness assessment in their countries and provide with corresponding evidence for their answers. Collected readiness scores were re-evaluated by the research team when conflicting answers existed between participants evaluating the same country. The final scores were determined based on provided evidence.

Second, participants took an online post-pilot assessment evaluating the tool followed on a five-point scale (strongly disagree, disagree, neutral, agree, and strongly agree) to determine the comprehensiveness, relevance, and acceptability of the tool. The feasibility of the tool was determined based on the ability to complete the readiness assessment and the ability to come up with one research idea that aimed to address identified problems based on the readiness scores. In addition, participants were also asked if they had any additional feedback to improve the abovementioned features and the tool as a whole. Based on the feedback, adaptation was followed to define the final version of the tool. Suggestions for major changes or inclusion of new items were inapplicable due to the extensive need for re-evaluation subsequently. Responses were collected *via* a Qualtrics survey (39).

## 3. Results

### 3.1. Phase 1: Tool development

#### 3.1.1. Development of the item pool and the first version of the tool

From the literature review, 16 tools and frameworks were identified. After the data extraction process, an item pool was formed that included 65 items (Supplementary material 1). The first version of the tool included eight and six items in the decision-making and implementation parts respectively. Table 1 provides a summary of the item generation process throughout the whole process, from the development of the item pool until the pilot test.

**Table 1:** Changes in the number of items during the item generation process of the country score tool.

**Step**	**Decision-making**					**Implementation**				
	**Start**	**Progress**			**Finish**	**Start**	**Progress**			**Finish**
		**Drop**	**Add**	**Edit**			**Drop**	**Add**	**Edit**	
Development of item pool^*^	38	0	0	38	8	27	0	0	38	6
Stakeholder consultation	8	0	1	8	9	6	0	0	6	6
Delphi round 1	9	0	1	7	10	6	0	1	6	7
Delphi round 2	10	0	0	5	10	7	0	1	4	8
Delphi round 3	10	1	0	0	9	8	0	0	0	8
Pilot testing	9	NA	NA	3	9	8	NA	NA	0	8

* In this step, items were dropped, edited or grouped together by the similarity leading to an increase in the number of items (Supplementary material 1).

NA, Not applicable.

#### 3.1.2. Stakeholder consultation

Eleven members participated in the plenary meeting. All proposed items were evaluated to be relevant and important with some suggestions for edits. Overall, it was suggested to specify which vaccines to be used as criteria to assess the strength of current national immunization programs. Furthermore, experts pointed out the importance of having guidelines or strategies based on local evidence in the country with regards to vaccine acceptability among aging adults. The suggestion led to adding one item to the decision-making part.

#### 3.1.3. Three-round Delphi study

The first round was completed by seven panelists. During the second and third rounds, all members completed the evaluation (*N* = 8). Description of the Delphi panel is presented in Table 2.

**Table 2:** Description of the Delphi panel.

Stakeholder group^*^	**Country**				
	**United Kingdom**	**Netherlands**	**Italy**	**Belgium**	**Hungary**
Ministry of health	x				
National Immunization Technical Advisory Group (NITAG) or equivalent	x	x	x	x	x
Health Technology Assessment Body (HTAB)	x		x		x
Representatives of: Healthy aging, General practitioners (GPs), pharmacists, nursing homes	x				x
Researchers	x		x		x
World Health Organization	x				

* Panelists might have expertise/experience in more than one stakeholder group.

Availability of national guidance or communication campaigns for generating acceptance and demand for vaccines in aging adults, together with availability of healthy aging strategies, were considered to be important by 100% of panelists in round 1. In contrast, availability of alternative measures for prevention and control of VPDs in aging adults was suggested to be dropped by 14% of panelists in round 1, subjected for re-evaluation in the next rounds, and finally excluded. Moreover, the panel suggested and agreed on adding three items related to (1) basic requirements for NITAGs (or equivalent), (2) hard-to-reach groups (e.g., illiterate individual and migrants), and (3) healthcare professionals' involvement in providing vaccine recommendations to aging adults. Thirteen items that did not reach the consensus in round 1 were re-evaluated over round 2 and/or round 3. Generally, the panel suggested using universal terms to optimize the transferability of the tool in different contexts, given the diversity in policy and organizational setup of immunization programs among European countries. Furthermore, suggestions for rewording were proposed by panelists with regards to comprehensiveness of the tool as a whole and scoring system of each item that can cover all possibilities to assess readiness.

### 3.2. Phase 2: Pilot testing

#### 3.2.1. Testing four features of the tool: Comprehensiveness, relevance, acceptability, and feasibility

Eight researchers participated and completed the readiness assessment and the post-pilot survey (Response rate 100%, *N* = 8). Table 3 shows the results of the post-pilot assessment in which a percentage of participants evaluating different features of the tool are presented.

**Table 3:** The post-pilot assessment result.

**Feature**	**Criteria**	**Results**			
		**Agree/ strongly agree**	**Neutral/ disagree/ strongly disagree**	**Yes**	**No**
Comprehensiveness	The tool covers all essential components	75.0%	25.0%		
	All components of the tool are well-explained	87.5%	12.5%		
Relevance	All components of the tool are relevant	100.0%	0.0%		
Acceptability	Time needed to complete the tool is acceptable	75.0%	25.0%		
	Use the tool or its results in the future			75.0%	25.0%
	Recommend the tool or its results to colleagues			100.0%	0.0%
Feasibility	Ability to complete the readiness assessment			100.0%	0.0%
	Ability to come up with a research plan after completing the readiness assessment			87.5%	12.5%

The tool was evaluated to be comprehensive to capture all essential aspects of the readiness assessment by 75% of participants. In addition, 88% of participants perceived all components well-explained. All participants rated the tool to be relevant in their countries. In terms of acceptability, 75% of participants indicated their acceptance with time required and the future use of the tool. Moreover, 100% of participants suggested that the tool and the readiness results would be useful to their colleagues in health economics, and other fields including public health, epidemiology, and health politics. The feasibility of the tool was accepted by more than 85% of participants.

#### 3.2.2. Final version of the tool

Participants of the pilot provided their feedback to redraft some of the sentences or provide more information for a better understanding on three items regarding existing vaccination programs (2 items) and healthy aging strategies. The final version of the tool consists of 17 items divided into decision-making and implementation parts. Each item has a guiding question, corresponding to explanations and rationales to inform assessment with readiness scores. Due to the large amount of information condensed in the tool, rationales and explanations of all items are presented separately in Supplementary material 2. The tool with guiding questions and readiness scores is presented in Table 4 of this manuscript.

**Table 4:** The final version of the country score tool (^*^) and readiness scores in four pilot countries.

**Domain**	**Guiding question**	**Readiness scores**	**NL**	**SER**	**HU**	**GER**
**Decision-making (9)**						
1. Surveillance of vaccine preventable diseases (VPD)	1. Is national surveillance system of vaccine preventable diseases in aging adults in place (e.g., influenza, pneumococcal, herpes zoster and pertussis diseases)?	0 = No national surveillance system exists for any disease 1 = One or two diseases have national surveillance systems 2 = Three or more diseases have national surveillance systems	2	2	2	2
2. Vaccine acceptability among aging adults	2.1. In the last 5 years, have any studies been conducted on the demand and acceptability toward recommended/implemented vaccines in your country?	0 = None at both local and national/state levels 1 = Some studies at local level and/or less than one study (per vaccine) at the national/state level 2 = At least one study (per vaccine) at the national/state level	2	1	1	1
	2.2. Is national guidance or communication campaign available for generating acceptance and demand for vaccines in aging adults in your country?	0 = No guidance/campaign at the national level available 1 = National guidance/campaign available which was developed based on theory or adaptation 2 = National guidance/ campaign available which was developed based on the country's evidence of acceptability and demand	1	0	1	1
3. Performance of existing immunization programs in aging adults	3.1. Did your country introduce any vaccines for aging adults into the national immunization program?	0 = No vaccine in aging adults was introduced 1 = One vaccine in aging adults was introduced 2 = Two or more vaccine in aging adults was introduced	2	0	1	2
	3.2. If the answer to the above question is “yes,” what are the vaccine coverage rates (VCRs) among aging population in the most recent year (according to the availability of data)?	0 = All VCRs < 25% OR the result is unknown OR the answer to question 3.1. is “No” 1 = VCRs ranges from 25 to 75% 2 = At least one vaccine reached VCR ≥ 75%	1	0	0	1
4. National immunization strategy	4. Is a National Immunization Strategy/Plan published which covered aging adult population?	0 = No immunization strategy publicly available 1 = Only pediatric immunization strategy publicly available 2 = National immunization strategy is published and covers both pediatric and adult vaccines	1	2	1	1
5. Stakeholders' involvement	5.1. Did your country establish a national immunization technical advisory group (or equivalent) that meets all basic World Health Organization (WHO) criteria in terms of membership and composition?	0 = No National immunization technical advisory group has been established 1 = National immunization technical advisory group has been established but has not met all criteria 2 = National immunization technical advisory group has been established and met all criteria	2	2	0	2
	5.2. Does national immunization technical advisory group (or equivalent) have adult vaccine workgroup(s)?	0 = No such working groups 1 = National immunization technical advisory group has no such working group, but is involved in other recommending bodies where vaccination in aging adults is approached and government is engaged 2 = Such working groups exist as part of a broader vaccine-specific working group or a standalone working group	0	0	0	2
6. The public health priority of diseases in aging adults	6. Is the publication of healthy aging strategies available in your country, and is immunization mentioned as a prevention measure?	0 = No healthy aging strategy publicly available 1 = Aging strategy available at the sub-national OR national level but does not mention adult vaccines 2 = Aging strategy available at the sub-national OR national level that mentions adult vaccines	1	1	1	2
**Implementation (8)**						
1. Vaccine financing	1.1. How many vaccines in aging adults have been recommended by the national immunization technical advisory group (or equivalent) in your country?	0 = None or one vaccine has been recommended 1 = Two vaccines have been recommended 2 = More than two vaccines have been recommended	2	1	0	2
	1.2. If the national immunization technical advisory group (or equivalent) in your country recommended vaccines for aging adults, how many of them have been implemented (full/partial reimbursement) within 5 years after the recommendation or up to the current moment (depends on which point comes first)?	0 = < 25% recommended vaccines have been implemented 1 = From 25 to 75% recommended vaccines have been implemented 2 = More than 75% recommended vaccines have been implemented	1	2	0	2
2. Advocacy	2. What is the level of governmental advocacy toward recommended vaccines for aging adults in the past 5 years in your country?	0 = No evidence of governmental advocacy toward recommended vaccines for aging adults 1 = At least one form of advocacy has been developed and involved one or two sectors 2 = At least one form of advocacy has been developed and involved multiple sectors (≥3) promoting aging adult vaccines	2	1	0	1
3. Access to vaccines	3. How easy it is to get vaccinated as an aging adult in your country in terms of location, provider, and requirement for vaccination appointment?	0 = Difficult to get vaccinated: only available at one provider with some limitations regarding required appointment or location 1 = Somewhat complicated: available at more than one provider (e.g., general practitioners—GPs, public health centers) but still some limitations regarding required appointment and location 2 = Easy to get vaccinated: available at GPs and/ or other providers (e.g., pharmacies, specialty physicians, long-stay facilities) without required appointment	1	2	1	2
4. Vaccine registry	4. What is the level of national/state vaccine registry for aging adults?	0 = No registry 1 = Sub-national or by individual health systems/providers/insurers 2 = Centralized	1	2	2	1
5. Active recommendation from healthcare professionals and reminder/recall centralization	5.1. What is the level of healthcare professionals' involvement in providing vaccine recommendations to aging adults and how is it monitored?	0 = No evidence of active involvement of healthcare professionals 1 = Active involvement of healthcare professionals is encouraged and monitored at sub-national level or by individual health systems/providers/ insurers 2 = Active involvement of healthcare professionals is encouraged and monitored at the national level	1	0	0	0
	5.2. What is the level of centralization of vaccine invite/reminder/recall in aging adults?	0 = No evidence: aging adults receive no reminder/recall 1 = Decentralized or mixture: some vaccinations have centralized reminder/recall, but some do not (aging adults only receive reminder/recall from healthcare professionals) 2= Centralized: auto-dial phone calls/ postcards/ text messages are provided for all recommended vaccines in aging adults	1	0	1	1
6. Hard-to-reach population	6. How much effort is being made to engage “hard-to-reach” aging adults (e.g., illiterates, migrants) to vaccinate?	0 = No additional effort at either local or national/state level 1 = Interventions/ strategies toward those groups have been implemented at local level but not yet at national/state level 2 = Interventions/strategies toward those groups have been implemented at national/state level	0	0	0	0

* This table only includes guiding questions and readiness scores in the tool; rationales and explanations of all items are presented separately in Supplementary material 2. NL, Netherlands; SER, Serbia; HU, Hungary; GER, Germany.

The readiness of countries with regards to decision-making is assessed by the availability of sufficient infrastructure, fully functioning supportive groups and relevant strategies. First, the national surveillance system of VPDs should be in place to provide baseline epidemiological data on VPDs as well as to measure the impact of vaccines on disease incidences, morbidity, and mortality. Moreover, previously successful vaccine introductions for aging adults are indicative of the relevant infrastructure of the NIP for this target population. Second, the involvement of relevant stakeholders is crucial for countries to make decisions on vaccine introductions. While a NITAG (or equivalent) is a basic need for an NIP, the availability of adult vaccine workgroups is a specific requirement for aging adult immunization. Such groups could be in place as standalone groups or initiated by involving relevant experts into recommending bodies where vaccination of aging adults is considered. Third, national immunization strategies and healthy aging strategies covering aging adult vaccination serve as a foundation to direct new vaccine introductions given the many health issues and resource constraints that need to be tackled. Additionally, communication strategies, adjusted by local evidence on vaccine demand and acceptability, are needed to ensure high vaccine uptake. If the evidence is not available, corresponding studies at the local or national level need to be conducted to guide policies.

In alignment with the decision-making part, items in the implementation part of the tool further assess how countries have proceeded with vaccine recommendations and identify how the current system can facilitate future vaccine adoption for aging populations. By reviewing vaccine financing aspects, decision-makers might consider if expanding immunization financing mechanisms would be needed for the long-term financial sustainability of the NIP. Furthermore, a high level of government advocacy toward recommended vaccines plays an important role in increasing vaccine uptake. Healthcare professionals (HCP) are essential stakeholders who are actively involved in the care of aging adults being influential in commencing and completing vaccination schedules. The active involvement of this group contributes to the success of the NIP by increasing the uptakes of not only newly introduced vaccines but also routine vaccines. Other aspects, including centralization of vaccine invite/reminder/recall, easy access to supplies of vaccines and vaccine registries should be in place. Extra effort and resources need to be allocated to engage “hard-to-reach” aging adults to be vaccinated. They are often at high-risk for infectious diseases but face access barriers to vaccination due to various reasons, for instance, distance from vaccination centers, healthcare provider discrimination, and legal restrictions.

#### 3.2.3. Readiness assessment results

Readiness scores for the four countries involved in the pilot testing are presented in Table 4. Although the scores cannot be considered as an official description of the readiness per country, the pilot assessment briefly reflects the current settings and performance of the national immunization programs in those countries. Overall, all countries demonstrated sufficient surveillance of at least three VPDs including influenza, pneumococcal disease, herpes zoster and/or pertussis. On the contrary, efforts to encourage hard-to-reach aging adults to be vaccinated were absent in all countries. In addition, availability of adult vaccine working groups and involvement of healthcare professionals were generally lacking. The Netherlands scored highly in items related to implemented vaccines including surveillance, local evidence of vaccine demand, and acceptability, the number of vaccines that have been introduced, and government advocacy to support vaccine introductions. However, supportive stakeholders, policies and strategies toward aging adult immunization programs were not fully in place. In contrast, Germany showed full preparations in those aspects which aligned with high scores in the item assessing the ease to vaccinate in the implementation part. Hungary was the only country of the four that did not have a NITAG. In addition, the country scored lowly in related items including having a national immunization plan, vaccine recommendations, and implementation in general. A similar pattern in implementation was seen in Serbia. There was no evidence on centralization of vaccine invite/reminder/recall in aging adults, together with active involvement of healthcare professionals. Although Serbia scored 0 in items related to existing immunization program and national guidance for communication, crucial stakeholders and national policy and surveillance systems were fully in place.

## 4. Discussion

This work has developed a tool to assess countries' readiness with regards to immunization programs in aging adults that can be used either before, or as part of the implementation process. Particularly, results from the readiness assessment can help to identify areas for improvement of the current programs as well as methodologies for future research to support decision-making and implementation of such programs in Europe.

In comparison with existing literature on this topic, our country score tool presents numerous unique features. First, unlike the purpose of other scoring systems such as archetype analysis aiming to categorize countries based on their characteristics (22), our tool serves as a readiness assessment which means that higher scores always indicate a higher level of readiness mentioned in the items. Second, although we propose a set of universal items that could be transferable among European countries, we consider each country to be unique. Thus, individual items in the tool are not necessarily equally important in all settings and subsequently no requisite labels are applied, which is opposite to the approach of a tool for planning and evaluating vaccination strategies in a published work (32). Additionally, the tool offers a great degree of flexibility for national authorities to specifically define the cut-offs of age for the national immunization programs considering the conceptualized cut-off of 50-year-old and over. Third, while some existing tools or guidance documents try to support countries in developing a comprehensive immunization program from zero (30, 41), our tool focuses on pinpointing important gaps in the current national immunization programs. By identifying those gaps, decision-makers can generate direct actions to strengthen current infrastructures and organizations such as setting up surveillance systems for common VPDs or forming working groups of aging adult vaccines. Fourth, aiming to not solely facilitate actions, our tool can help to go one step further to generate evidence needed for better decision-making. For that reason, the target audience of the tool is not only country-level decision-makers but also, researchers in different fields. Epidemiological research could be needed when countries score low in surveillance or having no evidence in monitoring vaccine coverage rates. In addition, when studies on vaccine demand and acceptability are lacking, behavioral research can help to generate tailored interventions or corresponding guidance to support implementation of the program. Furthermore, health economic research could be useful to address different gaps identified from the tool separately or simultaneously. For example, incorporating cost-effectiveness and value of information analyses can facilitate making well-informed policies regarding reimbursement of vaccination programs and setting priorities for future research.

In terms of content, our tool inherits from previous works to include well-known components aiming to support immunization programs; for instance: surveillance of VPDs, performance of existing programs, and public health priority of diseases (22). In addition, conducting research addressing the attitudes toward vaccines was considered crucial before the introduction of any vaccines to identify if low initial acceptance will exist or reasons for anti-vaccine sentiments (30). Nevertheless, it is important to turn knowledge into action. Therefore, one item was added to our tool to assess the availability of national guidance or communication campaigns for generating acceptance and demand for vaccines in aging adults. Regarding implementation, one novel item, relating to hard-to-reach populations, was suggested to be added and finally reached the consensus after the Delphi study. The item suggests to clearly define this population to inform strategy, planning, and resource determinations for target interventions to remove existing barriers to vaccinate (42). Besides confirmatory findings, contradictory judgments were found in this project. First, the item related to alternative measures for prevention and control of VPDs was excluded from our tool although it was considered as one of the criteria for decision-making in existing guidelines (30, 43). Those measures include, but are not limited to, treatment with antibiotics, antiviral therapies, or other medicines which could have a synergy with immunization programs. However, it might be irrelevant to include that aspect in the readiness assessment because those alternatives should be in place regardless of the availability of vaccines. Second, the involvement of healthcare workers by providing vaccine recommendation to aging adults was not initially included in the tool because of a non-significant association between this factor and vaccination coverage rates (VCR) of influenza in the elderly (44). However, considering that the success of one vaccination program is not necessarily comparable with others, the item was added by the Delphi panel.

Regarding the development process of the tool, the modified Delphi process provided many advantages. Firstly, the Delphi technique was an appropriate method when there was a lack of agreement, incomplete knowledge, uncertainty or lack of evidence (45). Compared to the conventional design, a modified Delphi approach allowed an active involvement of a steering group that performed a literature review in the problem area instead of open-ended discussion in the initial Delphi round (34). That helped to provide a comprehensive item pool for the following steps. Moreover, the internal VITAL stakeholder consultation was organized as an extra effort to ensure that comprehensiveness, relevance, and transferability of the tool were considered at the early phase of the development process. In addition, during the Delphi study, the use of a web-based platform allowed for anonymity and the inclusion of international experts. Additionally, predefined consensus criteria and a transparent procedure helped to reduce bias related to opinions of the steering group which was recognized as a disadvantage of the given study design (34). Furthermore, the comprehensiveness, relevance, and potential transferability of the tool were preliminary indicated in the pilot which has not been done in the development of existing tools. Finally, readiness scores of the four pilot countries showed that countries with higher scores including Germany and the Netherlands, had more vaccines being scheduled in the national immunization programs for aging adults, as well as higher vaccine coverage rates. That is indicative of the face validity of the tool.

Despite the thorough process, the study has some limitations which are primarily related to the small sample size in the Delphi study. There is no standard size of the panel members. It depends on the complexity of the topic and heterogeneity of the panel (34). With a similar topic, a Delphi panel of 8 participants was considered to be sufficient (32). The second limitation concerns the pilot testing with regards to the choice of countries, selection criteria for participants, and the sampling method. We selected four countries—partners of the VITAL project based on a convenience sampling approach. However, this set of four might be unrepresentative for European countries in general, and therefore might hamper the generalization of our findings. Regarding the selection criteria, although national authorities and researchers are both primary target audience of the tool, the inclusion of solely researchers can ensure the feasibility of the project but might affect the accuracy of the readiness assessment. Furthermore, the snowball sampling method could potentially introduce bias as the readiness assessment for one country might be performed by people working in the same organization. However, the difference between answers of two participants per country indicated that bias was unlikely to happen. The difference could be explained by the fact that not all of participants had strong background and experience in immunization. That suggests the ease to use of the tool when non-experts in the field could independently complete the tool with a reasonable level of accuracy. The third limitation comes with regards to the inclusions of items of the tool. Although the development process was evidence-based, the item inclusions could be subjective as indirect consequences of expert opinions from previous works and directly from our Delphi study. Therefore, it is uncertain that the effort spent to fill all gaps would always result in better decision-making. Future research should attempt to quantify the impact of individual items on the outcome of immunization programs measured by vaccine coverage rates. Besides, we suggest future research moving forward with the implication of the tool. First, it is important to conduct comprehensive field testing by using the tool in the appropriate target audience, being decision-makers, advisors and experienced researchers in immunization programs of aging adults across the majority of European countries. Second, digitalizing the tool could facilitate the usage of the tool on a large scale. Subsequentially, collection of readiness assessment data in many countries could help to quantify the association between readiness scores of individual item and vaccine coverage rates. Therefore, a precisely evidence-based readiness assessment could be developed to better inform decision-making at the national or even supranational level with regards to aging immunization programs.

## 5. Conclusion

Decision-making and implementation of immunization programs in aging adults in European countries need to be strengthened based on a holistic assessment and using relevant evidence. Our extensive consultations and iterative development process have resulted in an innovative tool that serves as a readiness assessment and simultaneously helps to identify methodologies for future research to support decision-making and implementation of such programs. The tool is considered to be comprehensive, relevant, acceptable, and feasible in four countries. This indicates the transferability and applicability of the tool in Europe. We suggest future effort of this work to focus on digitalizing and comprehensive field testing of the tool across the majority of Europeans countries. Moreover, additional studies should be conducted to quantify the impact of individual items in the readiness assessment on vaccine coverage rates.

## Data availability statement

The original contributions presented in the study are included in the article/Supplementary material, further inquiries can be directed to the corresponding authors.

## Ethics statement

According to the Dutch Law on Medical Research Involving Human Subjects (WMO), ethical approval is not required for research that does not involve participation by patients or use of patients' data (46). All expert participants gave informed consent before they took part in the study.

## Author contributions

TP conducted the literature review and wrote the first draft of the manuscript. JvdS, EB, and TP contributed to the development of the item pool, organized stakeholder consultation, Delphi study, and pilot test. MP has made a direct and intellectual contribution to the first version of the tool, and the stakeholder consultation. BN, TÁ, CW, DS, and NN substantially contributed to the final version of the tool. All authors read, edited, and approved the final manuscript.

## Supplementary material

Supplementary material 1 Literature review and the item pool.

Supplementary material 2 Supportive part of the country score tool.
