# Supplementary material for: Country score tool to assess readiness and guide evidence generation of immunization programs in aging adults in Europe
Source: Front Public Health. 2023 Jan 9;10:1080678. doi: 10.3389/fpubh.2022.1080678 (PMC9869118; doi:10.3389/fpubh.2022.1080678)
Supplement: Supplementary material 1 — Literature review and the item pool. [file Table_1.DOCX]

Supplementary Material 1

# Details of a literature review following JBI manual for Evidence synthesis - Scoping review

## Search strategies

Databases included MEDLINE (via PubMed), Scopus, and Institutional websites including World Health Organization (<https://www.who.int/publications/>) and European Centre for Disease Prevention and Control (<https://www.ecdc.europa.eu/en/publications-data>) following JBI Manual for Evidence Synthesis – Scoping review (1). Two sub-questions, to be answered by the review, were:

Sub-question 1: What country’s readiness assessment tools/frameworks for immunization programs ***in general populations*** exist?

Sub-question 2: What country’s readiness assessment tools/frameworks for immunization programs ***in adults*** exist?

Given the variability in use of the term “readiness assessment” in the literature, we considered all efforts that aimed to systematically assess a country’s ability to introduce vaccines to be relevant. Restrictions of articles that were published in English from 2010 to 2021 with the full text available were applied.

### Sub-question 1

What country’s readiness assessment tools and frameworks for immunization programs in **general population** are available?

Supplement Table 1: Inclusion/Exclusion Criteria of sub-question 1

|  | **Inclusion Criteria** | **Exclusion Criteria** |
| --- | --- | --- |
| Population (P) | Children or adolescents or adults (include maternal) | Minority (refugee, travelers, healthcare workers, HIV…) |
| Concept (C) | Comprehensive readiness assessment or strategy of national immunization programs, validated tool | - C1 (readiness assessment): experience from one specific country,  - C2 (immunization): mass immunization, supplement immunization program, specific vaccine including COVID-19 vaccines |
| Context (C) | National level | Global level, regional level, sub-nation levels |
| Types of evidence source | only systematic reviews and other types of reviews from PubMed | Full text in languages other than English or missing full text |

Supplement Table 2: Search strategy in PubMed (August 24, 2021)

| **Search** | **Query** |
| --- | --- |
| #1 | (Ready[tiab] OR readiness[tiab] OR Prepar*[tiab] OR Approach[tiab] OR Experience[tiab] OR Strateg*[tiab] OR Plan*[tiab] OR Practice[tiab]) |
| #2 | (Assess*[tiab] OR evaluate[tiab] OR Tool*[tiab] OR Scor*[tiab] OR Scale[tiab] OR Measur*[tiab] OR Instrument[tiab] OR Landscape[tiab] OR Manual[tiab] OR manuscript[tiab] OR checklist[tiab] OR framework[tiab]) |
| #3 | ("Immunization Programs"[Mesh] OR immunization*[tiab] OR immunisation*[tiab] OR "Vaccination"[Mesh] OR vaccin*[tiab]) NOT (papillomavirus[ti] OR HPV[ti]) |
| #4 | (implement*[tiab] OR introduc*[tiab] OR adopt*[tiab] OR deploy*[tiab]) |
| #5 | (Nation*[tiab] OR Countr*[tiab] OR “health system”[tiab] OR “healthcare system”[tiab] OR “public health”[tiab]) |
| #6 | ("Animals"[Mesh] NOT "Humans"[Mesh]) |
| #7 | ("Systematic Review"[pt] OR “systematic review”[tiab] OR systematic[sb] OR Review[pt] OR Review[tiab]) |
| #8 | #1 AND #2 AND #3 AND #4 AND #5 NOT #6 AND #7 |

Total number of retrieved articles: 1051

### Sub-question 2:

What country’s readiness assessment tools/frameworks for immunization programs in **adults** are available?

Supplement Table 3: Inclusion/Exclusion Criteria of sub-question 2

|  | **Inclusion Criteria** | **Exclusion Criteria** |
| --- | --- | --- |
| Population (P) | Only normal adults (including aging adults) | General population which covers other population along with adults, minority groups or adults with special condition/ health status (pregnancy, refugees, healthcare professionals, ….) |
| Concept (C) | Frameworks or tools of decision making and/or implementation for immunization programs in adults. | - C1 (readiness assessment): single aspect assessment (i.e. only vaccine supply, safety, communication, …);  - C2 (immunization): mass immunization, supplement immunization program, experience from a specific vaccine introduction in one specific country, COVID-19 vaccination program |
| Context (C) | National level | Global level, regional level, sub-nation levels |
| Types of evidence source | All types of publications including guidelines, tools, and reports from PubMed and Scopus | Full text in languages other than English or missing full text |

Supplement Table 4: Search code in PubMed (October 03, 2021)

| **Search** | **Query** |
| --- | --- |
| #1 | ("Adult"[Mesh] OR adult*[tiab] OR aging[tiab] OR ageing[tiab] OR elderl*[tiab] OR senior*[tiab] OR geriatri*[tiab] OR ((old[tiab] *OR* older[tiab]) *AND* (age[tiab] *OR* people[tiab] *OR* subject*[tiab] *OR* population*[tiab] *OR* person*[tiab])) OR septuagenarian*[tiab] OR octagenarian*[tiab] OR octogenarian*[tiab] OR nonagenarian*[tiab]) |
| #2 | (Ready[tiab] OR readiness[tiab] OR Prepar*[tiab] OR Approach[tiab] OR Experience[tiab] OR Strateg*[tiab] OR Plan*[tiab] OR Practice[tiab]) |
| #3 | (Assess*[tiab] OR evaluate[tiab] OR Tool*[tiab] OR Scor*[tiab] OR Scale[tiab] OR Measur*[tiab] OR Instrument[tiab] OR Landscape[tiab] OR Manual[tiab] OR manuscript[tiab] OR checklist[tiab] OR framework[tiab]) |
| #4 | ("Immunization Programs"[Mesh] OR immunization*[tiab] OR immunisation*[tiab] OR "Vaccination"[Mesh] OR vaccin*[tiab]) NOT (papillomavirus[ti] OR HPV[ti]) |
| #5 | (implement*[tiab] OR introduc*[tiab] OR adopt*[tiab] OR deploy*[tiab]) |
| #6 | (Nation*[tiab] OR Countr*[tiab] OR “health system”[tiab] OR “healthcare system”[tiab] OR “public health”[tiab]) |
| #7 | ("Animals"[Mesh] NOT "Humans"[Mesh]) |
| #8 | #1 AND #2 AND #3 AND #4 AND #5 AND #6 NOT #7 |

Filters: from 2010 to 2021

Total number of retrieved articles: 824

Supplement Table 5: Search code in Scopus of sub-question 2 (October 03, 2021)

| **Search** | **Query** |
| --- | --- |
| #1 | TITLE-ABS-KEY ( adult*  OR  ageing  OR  aging  OR  elderl*  OR  senior*  OR  geriatri* ) |
| #2 | TITLE-ABS-KEY ( ready  OR  readiness  OR  prepar*  OR  approach  OR experience OR  strateg* OR  plan* OR  practice ) |
| #3 | TITLE-ABS-KEY ( assess* OR evaluate OR tool* OR scor* OR scale OR measur* OR instrument OR landscape OR manual OR manuscript OR checklist OR framework ) |
| #4 | TITLE-ABS-KEY ( immunization OR immunisation OR vaccin* ) |
| #5 | TITLE-ABS-KEY ( implement* OR introduc* OR adopt* OR deploy* ) |
| #6 | TITLE-ABS-KEY ( nation* OR countr* OR "health AND system" OR "healthcare AND system" OR "public AND health" ) |
| #7 | #1 AND #2 AND #3 AND #4 AND #5 AND #6 |

Limit: from 2010, limit: English

Total number of retrieved articles: 1142

## Results

The review process is outlined in the Preferred Reporting Items for Systematic Reviews and Meta-Analyses (PRISMA) flow chart (***Figure 4***). We identified nine and five papers from sub-questions 1 and 2, respectively. In addition, three guidance documents from WHO, one report from ECDC websites, and one reference from included articles were identified by hand searching. In terms of the target population, four articles focus specifically on ageing populations, one on adults in general and fourteen on other populations including children, pregnant women, and non-specified populations.


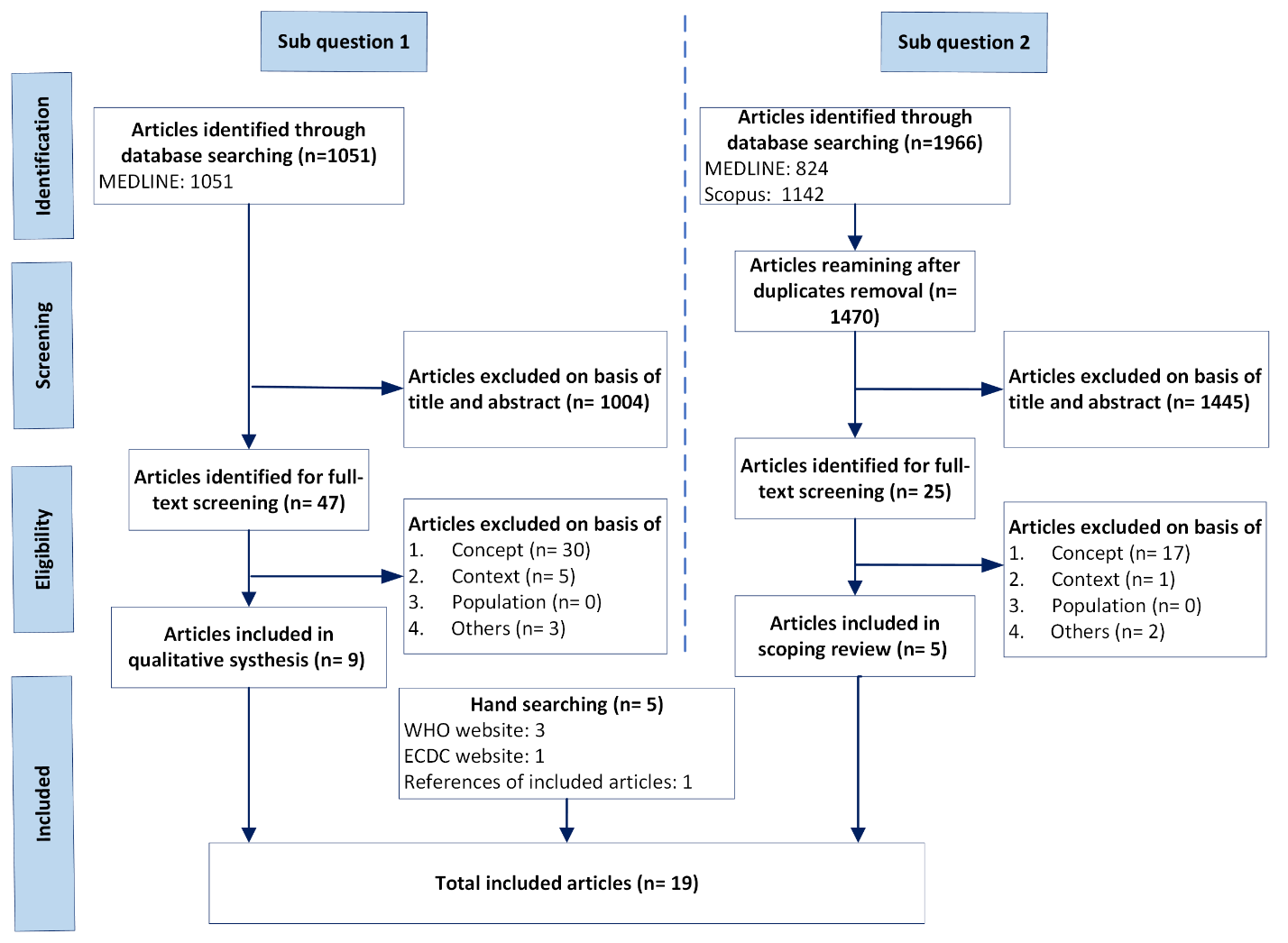


Supplement Figure 1. PRISMA flow chart of the literature review with two sub-questions

## Data extraction

In this step, we excluded three documents among 19 documents identified in the literature review from data analysis due to the upgrading to or being a part of other included articles.

Supplement Table 6: Data extraction of the literature review

| **Article** | **Author (Year)** | **Population** | **Aims/ purpose** | **Method** | **Reason for excluding** |
| --- | --- | --- | --- | --- | --- |
| Strategies for increasing uptake of vaccination in pregnancy in high-income countries: A systematic review | Biset et al. (2018) | Pregnancy | To identify effective strategies in increasing the uptake of vaccination in pregnancy in high-income countries and to make recommendations for England | A systematic review across six databases |  |
| National decision-making on adopting new vaccines: a systematic review | Burchett et al. (2012) | General | To identify the most appropriate ways to develop vaccine adoption decision-making, so as to improve decisions and, ultimately, health outcomes. | A systematic review across eight databases | There is an update systematic review of articles published from 2010-2020 (below) |
| National decision-making for the introduction of new vaccines: A systematic review, 2010–2020 | Donadel et al. (2021) | General | Guide discussions on evidence-informed immunization decision-making among country, sub-regional, and regional stakeholders. | A systematic review across six databases |  |
| Developing evidence-based immunization recommendations and GRADE | Duclos et al. (2012) | General | provide a process for reviewing evidence in the development of immunization recommendations and the integration of GRADE in this process | based on an evidence-based tool (GRADE) by extensive interactions with various experts at national (NITAGs) and supranational level (WHO, ECDC,…) | It becomes CAPACITI (see below) |
| Maternal immunization country readiness: a checklist approach | Giles ML, et al. (2020) | Pregnancy | This is a readiness checklist which enables countries to assess their capacity, strengths and weaknesses and identify a list of priorities to allow for smooth implementation of maternal vaccines. | Based on suggestions by a multidisciplinary expert group, a “checklist concept”, was drafted by the authors of this article and reviewed by regional and national experts at the WHO MIACSA (Maternal Immunization Antenatal Care Situational Analysis) Dissemination Meeting |  |
| Conceptual frameworks and key dimensions to support coverage decisions for vaccines | González-Lorenzo et al. (2014) | General | to support vaccine adoption and coverage decisions (provide vaccine free of charge) in a health system. | Three phases: 1. literature search; 2. synthesised the main dimensions and constructs suggested; 3. provided specific suggestions on target vaccine adoption decisions | Included in another systematic review - Donadel et al. (2021) |
| Vaccine strategies: Optimising outcomes | Hardt K, et al. (2016) | General | provide main elements of vaccination strategies | Not mentioned (the review was developed by expertise of authors using selected references) |  |
| Introduction of new vaccines for immunization in pregnancy – Programmatic, regulatory, safety and ethical considerations | Kochhar S, et al. (2019) | Pregnancy | provide a framework for the supportive data, policies, and activities needed to advance a vaccine program from product licensure to successful country-level implementation | Developed by a group of vaccine authorities based on recent vaccine adoptions and an existing set of WHO guidelines for new vaccine introduction. Reviewed by CDC reviewers |  |
| The first national adult immunization summit 2012: Implementing change through action | Shen et al. (2013) | Adult | provide potential participant-identified strategic tasks to improve vaccine coverage rate in adults | This is a Conference report based on a meeting with wide range of immunization partners in US |  |
| Situational assessment of adult vaccine preventable disease and the potential for immunization advocacy and policy in low- and middle-income countries | Sauer et al. (2021) | Adult | provide a broad overview of the key considerations regarding the need for and value of vaccination for older adults to advance adult immunization policies and advocacy | Conducted a literature review; summarized evidence to identify gaps and develop recommendations |  |
| Developing an Evidence-Based Tool for Planning and Evaluating Vaccination Strategies Aimed at Improving Coverage in Elderly and At-Risk Adult Population | Calabrò et al. (2021) | Elderly and at-risk adults | to help developing and appraising vaccination strategies in order to improve coverage in elderly and at-risk adult population | A scoping review and a Delphi study |  |
| Vaccination and healthy ageing: How to make life-course vaccination a successful public health strategy | Michel et al. (2010) | Aging adult | examines four common determinants for the successful implementation of 50+ vaccination programs and provide suggestions of actions to improve vaccine uptake | conducted a literature review to identify barriers (not in a systematic way); then provide suggestion accordingly |  |
| Archetype analysis of older adult immunization decision-making and implementation in 34 countries | Privor-Dumm et al. (2020) | Aging adult | provide 2 sets of indicators that most differentiated countries in terms of decision-making and implementation of adult immunization programs | mixed-methods: a landscape review of secondary data from 34 countries and 120 key informant interviews in six countries |  |
| Vaccination of 50+ adults to promote healthy ageing in Europe: The way forward | Esposito et al. (2018) | Aging adult | identify gaps and barriers in making vaccination of adults aged 50+ a reality and to agree upon further joint actions in Europe | based on a discussion of stakeholders in the area of vaccines and vaccination from 19 European Countries |  |
| Principles and considerations for adding a vaccine to a national immunization programme: From decision to implementation and monitoring | WHO (2014) | General | To assist countries in making informed decisions about adding a vaccine to a national immunization program; To guide the planning of a vaccine introduction; and To suggest ways to use the introduction of the vaccine to strengthen immunization and health systems. | update the WHO previous guideline, using recent research findings |  |
| WHO-UNICEF Guidelines for Comprehensive Multi-Year Planning for Immunization Update September 2013 | WHO (2013) | General | presents a series of steps to develop a comprehensive multi-year plan for immunization (cMYP), and includes both a planning tool and a costing tool | update WHO/UNICEF guideline (2005), using recommendations from stakeholders via consultations undertaken in 2011 - 2012 |  |
| Country-led Assessment for Prioritization in Immunization (CAPACITI): Decision-support framework | WHO (2021) | General | outlines the steps of a structured recommendation process | based on multi-criteria decision analysis (MCDA) |  |
| Key aspects regarding the introduction and prioritisation of COVID-19 vaccination in the EU/EEA and the UK | ECDC (2020) | General | provides an overview of the key aspects related to the initial phases following the introduction of a new vaccine into national vaccination schedules. | Not mentioned |  |
| Informing vaccine decision-making: A strategic multi-attribute ranking tool for vaccines—SMART Vaccines 2.0 | Knobler et al. (2017) | General | to support decision-making among multiple stakeholders in the process of prioritizing investments to optimize the outcomes of vaccine development and deployment | Not mentioned |  |

# Item pool

Supplement Table 7: Item pool

| **Components** | **Sub-components** | Donadel et al. (2021) (2) | WHO (2014) (3) | Giles et al. (2020) (4) | WHO-UNICEF 2013 (5) | WHO (2021) (6) | Knobler et al. (7) | ECDC (2020) (8) | Biset et al. (2018) (9) | Hardt et al. (2016) (10) | Kochhar et al. (2019) (11) | Shen et al. (2013) (12) | Sauer et al. (2021) (13) | Calabrò et al. (2021) (14) | Privor-Dumm et al. (2020) (15) | Michel et al. (2010) (16) | Esposito et al. (2018) (17) |
| --- | --- | --- | --- | --- | --- | --- | --- | --- | --- | --- | --- | --- | --- | --- | --- | --- | --- |
| **I. Decision-making of a vaccine introduction** | | | | | | | | | | | | | | | | | |
| 1. The importance of the health problem | Burden of disease (e.g. prevalence) | x | x | x |  |  | x |  |  | x | x |  | x |  | x |  |  |
|  | Political priority | x | x | x |  |  |  |  |  |  |  |  |  |  | x |  |  |
|  | Costs of disease | x | x | x |  |  |  |  |  |  |  |  | x |  |  |  |  |
|  | Perceptions of importance (e.g. in terms of perceived severity or vulnerability) | x | x | x |  |  |  |  |  |  |  |  |  |  |  |  |  |
|  | Other | x |  |  |  |  |  |  |  |  |  |  |  |  |  |  |  |
| 2. Vaccine characteristics | Efficacy/effectiveness | x | x |  |  |  | x |  |  |  | x |  |  |  |  |  |  |
|  | Vaccine safety | x | x |  |  |  | x |  |  |  | x |  |  |  |  |  |  |
|  | Delivery issues (e.g. vaccine schedule) | x | x | x |  |  | x |  |  |  |  |  |  |  |  |  |  |
|  | Other characteristics (storage and preparation,..) | x | x |  |  |  | x |  |  |  |  |  |  |  | x |  |  |
| 3. Programmatic considerations | Feasibility of delivering immunizations | x | x | x |  |  | x |  |  | x | x |  | x |  |  |  |  |
|  | Vaccine supply | x | x |  |  |  | x |  |  | x | x |  |  |  |  |  |  |
|  | Current immunization programs (IPs) |  | x | x |  |  | x |  |  | x |  |  | x |  |  |  |  |
|  | Recent IPs in this population |  |  | x | x |  | x |  |  |  |  |  |  |  | x |  |  |
|  | Strengths of health system toward target population |  |  | x | x |  | x |  | x |  | x |  |  |  | x | x |  |
|  | vaccine recommendation |  |  |  |  |  |  |  |  |  | x |  |  |  |  |  |  |
|  | Encourage life-course vaccination to promote healthy ageing |  |  |  |  |  |  |  |  |  |  |  |  |  |  | x |  |
| 4. Acceptability |  | x | x | x |  |  |  |  |  |  | x |  |  |  |  |  |  |
| 5. Accessibility, equity and ethics |  | x | x |  |  |  |  | x |  |  |  |  |  |  |  |  |  |
| 6. Financial/economic issues | Economic evaluation | x | x |  |  |  |  |  |  |  | x |  | x |  |  |  |  |
|  | Incremental costs | x | x |  |  |  |  |  |  |  |  |  |  |  |  |  |  |
|  | Funding sources | x | x |  |  |  |  |  |  |  | x |  | x |  |  |  |  |
|  | Vaccine price | x | x |  |  |  |  |  |  |  |  |  |  |  |  |  |  |
|  | Financial sustainability | x | x | x |  |  |  |  |  | x | x |  | x |  |  |  |  |
|  | Other (including affordability) | x | x |  |  |  |  |  |  |  |  |  |  |  |  |  |  |
| 7. Impact of vaccination | Impact on health outcomes | x | x |  |  |  | x |  |  |  |  |  | x |  |  |  |  |
|  | Impact on non-health outcomes | x | x |  |  |  | x |  |  |  |  |  | x |  |  |  |  |
|  | Effect of co-administration | x | x |  |  |  | x |  |  |  |  |  |  |  |  |  |  |
|  | Risks of serotype replacement | x | x |  |  |  | x |  |  |  |  |  |  |  |  |  |  |
|  | Other impact | x | x |  |  |  | x |  |  |  |  |  | x |  |  |  |  |
| 8. Consideration of alternative interventions | Cost-effectiveness of alternatives | x | x |  |  |  |  |  |  |  |  |  |  |  |  |  |  |
|  | Effectiveness of alternatives | x | x |  |  |  |  |  |  |  |  |  |  |  |  |  |  |
|  | Other considerations | x | x |  |  |  |  |  |  |  |  |  |  |  |  |  |  |
| 9. Decision-making process | Evidence sources/quality of evidence | x | x |  |  | x | x | x |  |  |  |  | x |  |  |  | x |
|  | Stakeholders involved | x | x | x |  | x |  |  |  | x |  |  |  | x |  |  | x |
|  | availability of working group |  |  |  |  |  |  |  |  |  |  |  |  | x | x |  |  |
|  | Criteria and priority setting for decision-making |  |  |  |  | x | x |  |  |  |  |  | x |  |  |  |  |
|  | Procedures | x | x |  |  | x |  |  |  |  |  |  |  |  |  |  |  |
|  | Cues to action (e.g. disease outbreaks) | x |  |  |  |  |  |  |  |  |  |  |  |  |  |  |  |
| **II. Implementation** | | | | | | | | | | | | | | | | | |
| 1. Planning for a successful vaccine introduction | Updating immunization plans and policies and integrating them with the national health plan |  |  |  | x |  |  | x |  | x | x |  |  | x |  |  |  |
|  | Developing a vaccine introduction plan |  |  |  | x |  |  | x |  | x |  |  |  | x | x | x | x |
|  | Obtaining informed consent to vaccination |  |  |  |  |  |  |  |  |  |  |  |  | x |  |  |  |
|  | Centralization of adult Vaccine delivery |  |  |  |  |  |  |  |  |  |  |  |  |  | x |  |  |
|  | Centralization of health system delivery |  |  |  |  |  |  |  |  |  |  |  |  |  | x |  |  |
| 2. Choosing the immunization strategy | Phased or simultaneous introduction |  |  |  |  |  |  |  |  |  |  |  |  |  |  |  |  |
|  | Deciding whether to introduce more than one vaccine at a time |  |  |  |  |  |  |  |  |  |  |  |  |  |  |  |  |
|  | Optimal schedule for routine immunization and eligibility |  |  |  |  |  |  |  |  |  |  |  |  | x |  |  |  |
|  | Catch-up and supplemental immunization campaigns |  |  |  |  |  |  |  |  |  |  |  |  |  |  |  |  |
|  | New delivery strategies |  |  |  |  |  |  |  |  |  |  | x | x |  |  | x |  |
|  | Using the opportunity of a new vaccine introduction to implement integrated approaches towards disease control and health promotion |  |  |  |  |  |  |  |  |  |  |  |  |  |  |  |  |
| 3. Selecting the vaccine, presentation and formulation |  |  |  |  |  |  |  |  |  |  |  |  |  |  |  |  |  |
| 4. Assuring quality and procuring the vaccine and injection supplies |  |  |  |  | x |  |  | x |  |  |  |  |  | x |  |  |  |
| 5. Determining vaccine management, cold chain and logistics needs |  |  |  |  | x |  |  | x |  |  |  |  |  | x |  |  |  |
| 6. Ensuring injection safety and safe waste disposal |  |  |  |  | x |  |  |  |  |  |  |  |  | x |  |  |  |
| 7. Training and supervision of health personnel |  |  |  |  | x |  |  |  | x | x |  | x |  | x |  |  | x |
| 8. Advocacy, communications and social mobilization | Advocacy, communication, social mobilization |  |  | x | x |  |  | x | x | x |  | x | x | x | x | x | x |
|  | Empowering providers |  |  |  |  |  |  |  |  |  |  | x |  |  |  |  |  |
|  | Improve the involvement of HCPs |  |  |  |  |  |  |  |  |  |  |  |  |  |  | x |  |
| 9. Updating information systems |  |  |  | x | x |  |  |  | x |  |  |  |  | x | x | x |  |
| 10. Access to vaccine |  |  |  |  |  |  |  |  |  |  |  |  |  |  | x |  |  |
| 11. Coverage monitoring |  |  |  |  |  |  |  | x |  | x | x |  | x |  | x |  |  |
| 12. Disease surveillance |  |  |  |  |  |  |  | x |  | x | x |  | x |  |  |  |  |
| 13. Vaccine safety monitoring |  |  |  |  |  |  |  | x |  |  | x |  | x |  |  |  |  |
| 14. Assessing program implementation and lessons learned | Post-introduction evaluations |  |  |  |  |  |  | x |  |  | x |  |  |  |  |  |  |
|  | Performance and quality measures of healthcare provider |  |  |  |  |  |  |  |  |  |  | x |  |  |  |  |  |
|  | monitoring of vaccine acceptability and behavioral research |  |  |  |  |  |  | x |  |  |  |  |  |  |  |  |  |

**References**

1. Peters MDJ, Marnie C, Tricco AC, Pollock D, Munn Z, Alexander L, et al. Updated methodological guidance for the conduct of scoping reviews. JBI evidence synthesis. 2020;18(10):2119-26.

2. Donadel M, Panero MS, Ametewee L, Shefer AM. National decision-making for the introduction of new vaccines: A systematic review, 2010-2020. Vaccine. 2021;39(14):1897-909.

3. World Health Organization. Principles and considerations for adding a vaccine to a national immunization programme: from decision to implementation and monitoring. Geneva: World Health Organization; 2014.

4. Giles ML, Mason EM, Lambach P, Mantel C. Maternal immunization country readiness: a checklist approach. Human vaccines & immunotherapeutics. 2020;16(12):3177-83.

5. World Health Organization. WHO-UNICEF guidelines for comprehensive multi-year planning for immunization: update September 2013. Geneva: World Health Organization; 2014. Contract No.: WHO/IVB/14.01.

6. World Health Organization. Country-led Assessment for Prioritization in Immunization (CAPACITI): Decision-support framework Version 2.1. 2021 November 18, 2020.

7. Knobler S, Bok K, Gellin B. Informing vaccine decision-making: A strategic multi-attribute ranking tool for vaccines-SMART Vaccines 2.0. Vaccine. 2017;35 Suppl 1:A43-a5.

8. European Centre for Disease Prevention and Control. Key aspects regarding the introduction and prioritisation of COVID-19 vaccination in the EU/EEA and the UK. 26 October 2020. ECDC: Stockholm; 2020.

9. Bisset KA, Paterson P. Strategies for increasing uptake of vaccination in pregnancy in high-income countries: A systematic review. Vaccine. 2018;36(20):2751-9.

10. Hardt K, Bonanni P, King S, Santos JI, El-Hodhod M, Zimet GD, et al. Vaccine strategies: Optimising outcomes. Vaccine. 2016;34(52):6691-9.

11. Kochhar S, Edwards KM, Ropero Alvarez AM, Moro PL, Ortiz JR. Introduction of new vaccines for immunization in pregnancy - Programmatic, regulatory, safety and ethical considerations. Vaccine. 2019;37(25):3267-77.

12. Shen AK, Bridges CB, Tan L. The first national adult immunization summit 2012: Implementing change through action. Vaccine. 2013;31(2):279-84.

13. Sauer M, Vasudevan P, Meghani A, Luthra K, Garcia C, Knoll MD, et al. Situational assessment of adult vaccine preventable disease and the potential for immunization advocacy and policy in low- and middle-income countries. Vaccine. 2021;39(11):1556-64.

14. Calabrò GE, Carini E, Tognetto A, Mancinelli S, Sarnari L, Colamesta V, et al. Developing an Evidence-Based Tool for Planning and Evaluating Vaccination Strategies Aimed at Improving Coverage in Elderly and At-Risk Adult Population. Frontiers in public health. 2021;9:658979.

15. Privor-Dumm L, Vasudevan P, Kobayashi K, Gupta J. Archetype analysis of older adult immunization decision-making and implementation in 34 countries. Vaccine. 2020;38(26):4170-82.

16. Michel JP, Gusmano M, Blank PR, Philp I. Vaccination and healthy ageing: How to make life-course vaccination a successful public health strategy. European Geriatric Medicine. 2010;1(3):155-65.

17. Esposito S, Principi N, Rezza G, Bonanni P, Gavazzi G, Beyer I, et al. Vaccination of 50+ adults to promote healthy ageing in Europe: The way forward. Vaccine. 2018;36(39):5819-24.
