# Supplementary material for: Country score tool to assess readiness and guide evidence generation of immunization programs in aging adults in Europe
Source: Front Public Health. 2023 Jan 9;10:1080678. doi: 10.3389/fpubh.2022.1080678 (PMC9869118; doi:10.3389/fpubh.2022.1080678)
Supplement: Supplementary material 2 — Supportive part of the country score tool. [file Table_2.DOCX]

Supplementary Material 2

Supplement Table 8: Supportive part of the country score tool

| **Domain** | **Definition/ Explanation** | | **Rationale** |
| --- | --- | --- | --- |
| **Decision-making** | |  | |
| 1. Surveillance of vaccine preventable diseases (VPD) | Surveillance is an ongoing, systematic process of information collection, analysis, interpretation, visualization, dissemination, and connection to public health programs. Surveillance systems are organized networks of people and activities dedicated to managing and maintaining surveillance for specific conditions. Surveillance systems can operate at various levels within countries, from local to national, or on a global scale (1). | | Surveillance provides baseline data - To measure the impact of the vaccine on disease incidence, morbidity (hospitalization, disability rate) and mortality (2) - To monitor other epidemiological changes in the disease, such as shifts in the age patterns or in the types or sub-types of the organism, as well as to detect outbreaks (2) It is recommended to conduct surveillance for influenza and pneumococcal diseases due to the high burden on the elderly (3, 4). In addition, surveillance of herpes zoster disease (HZ) is recommended if countries consider introducing HZ vaccines, and pertussis surveillance is recommended for outbreak detection (5, 6) |
| 2. Vaccine acceptability among ageing adults | Active demand represents intentional seeking of vaccination by an informed individual who perceives benefits and need of a particular vaccination. Vaccine acceptability (or ‘acceptance’) refers to the degree to which an individual is willing to undergo the recommended course of vaccination (7) Due to the impact of COVID-19 in all aspects of healthcare and research, a five-year window excluding COVID era should be taken. | | To ensure a successful vaccine introduction, it can be critical to assess the current climate towards vaccines in the country, the potential impact of the vaccine introduction on the uptake of other vaccines in the program, and of the health sector’s ability to develop strategies or interventions to prevent negative perceptions from derailing the new vaccine introduction (2) A new vaccine in high demand may bring previously unimmunized people into the clinic, resulting in increases in coverage of all routine vaccines. On the other hand, low acceptability can reduce immunization coverage (2) |
|  | Guidance and communication campaigns are tools designed for national immunization program managers, partner organizations, implementers and civil society representatives for community engagement and managing misinformation. | | Safety concerns about new vaccines, the rise of anti-vaccine movements and misinformation have resulted in low initial acceptance. Thus, to ensure a successful introduction, it can be critical to develop context-specific strategies or interventions to prevent negative perceptions (2) |
| 3. Performance of existing immunization programs in ageing adults | National immunization programs are responsible for the management of immunization at the country level and cover a range of functions from establishing evidence-based policies to financing and procurement of vaccines, vaccine management and logistics, delivery of vaccination services and collection, as well as analysis and use of immunization data (8). | | Recent experience introducing a vaccine in adults may mean that relevant mechanisms, e.g., healthcare worker training programs, communication strategies etc. have been developed and strengthened in the National Immunization Program (NIP) in addition to strengthening surveillance systems and monitoring and evaluation processes (9). |
|  | World Health Organization and European Council recommended to attain influenza vaccine coverage of 75% among older people (10). For other diseases, there is no official recommendation on coverage rate. The United States aims to increase pneumococcal VCR to 90% in all people aged ≥ 65 years (11). Along with reaching the pre-specified VCR, countries should also aim to increase absolute VCRs. | | Reviewing VCRs could help to identify which immunization program performs well and which program does not. By doing so, one could identify lessons learned which can support the implementation plan. |
| 4. National immunization strategy | The National Immunization Strategy (NIS) is a streamlined planning document that focuses on a strategic period of 5 years (12). The NIS defines:  1. The immunization vision to be achieved over the long term (generally 10 years).  2. Specific objectives to be achieved at the end of the strategic period (5 years).  3. Priority strategies, consisting of a costed set of interventions to achieve the objectives, as well as the measures to mitigate against risks associated with the selected interventions (12). | | National Immunization plan articulates a comprehensive strategy to enhance all aspects of vaccination (13).  Despite robust child immunization programs in most countries, adult immunization programs and policies lag far behind (14). To address the persistently low vaccination coverage rates among adults, it is recommended to the country to develop National Adult Immunization strategy (15). |
| 5. Stakeholders involved | National Immunization Technical Advisory Group (NITAG) is a technical resource supplying guidance to national policy makers and program managers to enable them to make evidence-based immunization related policy and program decisions (16). Synonyms for NITAG include National Advisory Committee on Immunization or National Committee on Immunization Practice. We use the term NITAG throughout this document. WHO's guidance for establishment of such group is available (16). | | NITAG is particularly important in view of the complex and vast bodies of evidence and the global interdependence and integration of health systems |
|  | An adult vaccine workgroup is a diverse multi-sectoral partnership that strives to improve vaccination coverage rates and adult vaccination best practices through systems-level approaches. The workgroup includes public health experts, medical center occupational health directors, state and local health departments, the private medical sector, vaccine manufacturers and distributors, academic institutions, and medical professional organizations. | | A workgroup takes specific actions that will lead to improvements in vaccine uptake, such as through reducing barriers for payment, increasing access to vaccines and vaccinators, and raising awareness of adult immunization recommendations |
| 6. The public health priority of diseases in ageing adults | This document sets the strategic direction for the next period (could be 10 years) for the delivery of services to ageing population. The strategies and detailed action plan are developed through extensive collaboration with the health and social sectors; and are usually aligned to the World Health Organization’s Global strategy and national action plan on health (17) | | Given the many health issues and resource constraints that each country faces, health policymakers need to set priorities on which health problems to address and what specific interventions to consider for ageing adults (2). If combatting vaccine preventable diseases in ageing adults is considered as a public health priority, immunization programs should be considered. |
| **Implementation** | | | |
| 1. Vaccine financing | Recommendations by National Immunization Technical Advisory Group (NITAG) for national immunization programs are based on clinical, economic, or public health factors in most European countries (18). Currently COVID-19 vaccines are not considered in this question. | | Vaccination against influenza and Streptococcus pneumoniae is usually recommended in European countries. Some countries also recommend vaccination against herpes zoster for older adults (19). The recommendation of NITAG is key feature that leads to Final Decision and National immunization program inclusion is usually made by the Ministry of Health (18) |
|  | In most of European countries, the National immunization technical advisory group (NITAG) recommends inclusion of a vaccine in the National immunization program and makes recommendations for funding. A final decision regarding the national immunization program is usually made by the Ministry of Health. The decision on introducing a vaccine does not necessarily mean providing a vaccine free of charge, depending on the country (18). | | Providing vaccine free of charge is considered as a key element for a successful immunization program (20). It is recommended for decision-makers to assess the feasibility of expanding immunization financing mechanisms (14). High percentage of funded vaccines can reflect the long-term financial sustainability of the country for immunization programs |
| 2. Advocacy | Advocacy is characterized as any effort to influence policy and decision makers, to fight for social change, to transform public perceptions and attitudes, to modify behaviors, or to mobilize human and financial resources (21). Advocacy can take many forms, e.g., awareness raising, communications and media work; communication for behavior change; Developing partnerships/ coalitions/ alliances; Research/ publications (22). This question refers to any initiatives/ strategies that have been done by governmental agencies. Sectors are defined as four categories: (i) Potential partners: aid organizations, nongovernmental organizations, researchers, and others who have a stake in immunization; (ii) Policy and decision makers who influence immunization (in hospitals, research institutions, businesses, NGOs, ...); (iii) The general public; (iv) Mass media.  An example of governmental advocacy: in the Netherlands, an infographic about pneumococcal vaccination that is updated annually by RIVM in collaboration with other stakeholders is printed on the back of the invitation letter to ageing adults. | | Government agencies are important advocates for immunization programs (21). Besides providing financial resource for recommended vaccines, government agencies play an important part in bring on board different stakeholders so support implementation of the immunization program. After a vaccine is introduced, continued advocacy efforts can help achieve and maintain coverage levels, community demand and can communicate the health gains and benefits achieved. Effective advocacy may increase the visibility of a topic and help facilitate dialogue (22) |
| 3. Access to vaccines | The ease of getting vaccinated can be assessed by measuring facilitation factors, such as the number of immunization providers, and whether appointment is required. It is recommended to expand access to vaccinations to non-medical places (e.g., pharmacies), particularly in the context of public health emergencies (e.g., COVID-19), more innovative delivery should be explored (e.g., self-administration of mobile outreach) (14) | | Access to immunization is known to influence older adult immunization uptake (23). Lower uptake may be due to limited mobility, health system complexity, limited places, or providers of adult vaccination (23). |
| 4. Vaccine registry | Vaccine registry (Immunization information system - IIS) is a confidential, population-based, computerized database that records all immunization doses administered by participating providers to persons residing within a given geopolitical area (24). | | Scale of registry operation (regional, national, etc.) contributes to performance of immunization programs in older adults (23)  At the point of clinical care, an IIS can provide consolidated immunization histories for clinical decision makers. At the population level, it provides aggregate data on vaccinations for use in surveillance and program operations, and in guiding public health action with the goals of improving vaccination rates and reducing vaccine-preventable disease (24) |
| 5. Active recommendation from healthcare professionals and reminder/recall centralization | Active involvement of healthcare professionals (HCPs) such as clinicians, pharmacists, and clinical subspecialists is described as: - Assess the vaccination status of people  - Identify vaccines patients need, then clearly recommend needed vaccines. - Offer needed vaccines or refer people to another provider for vaccination. Depending on country's specific context, encouragement HCPs to recommend vaccines could be stated in the national immunization plan/strategy, on the national website or/and in the organization's policy. Involvement of HCPs can be monitored/ evaluated by monitoring vaccination rates within general practice (25) or by utilizing immunization information system (IIS). | | HCPs are key players in the implementation of vaccination programs and can change the perceptions of vaccination and thus improve uptake (25). |
|  | Centralized reminder/recall involves notifying individuals when vaccinations are due (reminders) or late (recalls), and enables messages to be sent via mail, auto-dial call, text, or other method across large geographic areas and to be targeted and tailored to the population (26). | | Reminder/ recall for vaccines can increase vaccination rates (27). However, evidence in children shows that less than one-fifth of practices send reminders routinely (28). Barriers include limited resources, technological challenges, and competing priorities. Centralized reminder/recall is a potential solution. It was effective at increasing influenza vaccination rates in adults aged ≥65 years over a short period, without burdening the practices, and at a reasonable cost (29). |
| 6. Hard-to-reach population | Hard-to-reach populations are those who face supply-side barriers to vaccination due to geography by distance or terrain, transient or nomadic movement, healthcare provider discrimination, lack of healthcare provider recommendations, inadequate vaccination systems, war and conflict, home births or other home-bound mobility limitations, or legal restrictions (30).  Supply-side interventions should be considered to engage "hard-to-reach" population (30). Pharmacists could potentially contribute to supply-side interventions, as they are established advocates, educators as well as qualified providers of vaccinations (31). | | Hard-to-reach populations are often at high-risk for infectious diseases (30). Defining these populations clearly is essential for policy makers, governments, donors, and the vaccine community to inform strategy, planning, and resource determinations for target interventions to remove existing barriers to vaccination. In general, additional efforts and resources need to be allocated to increase immunization coverage among this group (31). |

**References**

1. Detels R, Gulliford M, Karim QA, Tan CC. Oxford Textbook of Global Public Health: Oxford University Press; 2015 2015-02.

2. World Health Organization. Principles and considerations for adding a vaccine to a national immunization programme: from decision to implementation and monitoring. Geneva: World Health Organization; 2014.

3. World Health Organization. Influenza. Surveillance standards for vaccine-preventable diseases - Influenza. 2nd ed. Geneva: World Health Organization; 2018.

4. World Health Organization. Pneumococcus. Surveillance standards for vaccine-preventable diseases - Influenza. 2nd ed. Geneva: World Health Organization; 2018.

5. World Health Organization. Varicella. Surveillance standards for vaccine-preventable diseases - Influenza. 2nd ed. Geneva: World Health Organization; 2018.

6. World Health Organization. Pertussis. Surveillance standards for vaccine-preventable diseases - Influenza. 2nd ed. Geneva: World Health Organization; 2018.

7. Nadarzynski T, Frost M, Miller D, Wheldon CW, Wiernik BM, Zou H, et al. Vaccine acceptability, uptake and completion amongst men who have sex with men: A systematic review, meta-analysis and theoretical framework. Vaccine. 2021;39(27):3565-81.

8. Cherian T, Mantel C. National immunization programmes. Bundesgesundheitsblatt, Gesundheitsforschung, Gesundheitsschutz. 2020;63(1):16-24.

9. Giles ML, Mason EM, Lambach P, Mantel C. Maternal immunization country readiness: a checklist approach. Human vaccines & immunotherapeutics. 2020;16(12):3177-83.

10. European Centre for Disease Prevention and Control. Seasonal influenza vaccination in Europe. Vaccination recommendations and coverage rates in the EU Member States for eight influenza seasons: 2007–2008 to 2014–2015. Stockholm: ECDC; 2017.

11. U.S. Department of Health and Human Services. Immunization and Infectious Diseases [cited March 01, 2022 ]. IID-13.1Increase the percentage of noninstitutionalized adults aged 65 years and older who are vaccinated against pneumococcal disease]. Available from: <https://www.healthypeople.gov/2020/topics-objectives/topic/immunization-and-infectious-diseases/objectives>.

12. World Health Organization. Guidelines for developing a national immunization strategy: frequently asked questions, key concepts and terms. Geneva: World Health Organization; 2021 2021. Contract No.: WHO/IVB/2021.05.

13. U.S. Department of Health & Human Services. U.S National Vaccine Plan 2010: Protecting the Nation’s Health through Immunization. Washington, DC.2010.

14. Sauer M, Vasudevan P, Meghani A, Luthra K, Garcia C, Knoll MD, et al. Situational assessment of adult vaccine preventable disease and the potential for immunization advocacy and policy in low- and middle-income countries. Vaccine. 2021;39(11):1556-64.

15. U.S. Department of Health and Human Services. Vaccines National Strategic Plan 2021–2025. Washington, DC.; 2021.

16. Duclos P. National Immunization Technical Advisory Groups (NITAGs): guidance for their establishment and strengthening. Vaccine. 2010;28 Suppl 1:A18-25.

17. New Zealand Ministry of Health. Healthy Ageing Strategy 2021 [updated September 05, 2021; cited March 01, 2022 ]. Available from: <https://www.health.govt.nz/our-work/life-stages/health-older-people/healthy-ageing-strategy-update#:~:text=New%20Zealand%E2%80%99s%20Healthy%20Ageing%20Strategy%20was%20released%20in,a%20respectful%20end%20of%20life%20in%20age-friendly%20communities>.

18. Laigle V, Postma MJ, Pavlovic M, Cadeddu C, Beck E, Kapusniak A, et al. Vaccine market access pathways in the EU27 and the United Kingdom - analysis and recommendations for improvements. Vaccine. 2021;39(39):5706-18.

19. Weinberger B. Vaccines for the elderly: current use and future challenges. Immunity & ageing : I & A. 2018;15:3.

20. Calabrò GE, Carini E, Tognetto A, Mancinelli S, Sarnari L, Colamesta V, et al. Developing an Evidence-Based Tool for Planning and Evaluating Vaccination Strategies Aimed at Improving Coverage in Elderly and At-Risk Adult Population. Frontiers in public health. 2021;9:658979.

21. Lasher H. Advocacy for Immunization: How to generate and maintain support for vaccination programs: GAVI Alliance/PATH; 2001.

22. ANA F. CARVALHO. Advocacy for Stronger Immunization Programs: Sabin Vaccine Institute; 2018 [Available from: <https://www.sabin.org/sites/sabin.org/files/carvalho_rev7.20.18.pdf>.

23. Privor-Dumm L, Vasudevan P, Kobayashi K, Gupta J. Archetype analysis of older adult immunization decision-making and implementation in 34 countries. Vaccine. 2020;38(26):4170-82.

24. National Center for Immunization and Respiratory Diseases. Immunization Information Systems 2019 [updated June 7, 2019; cited February 01, 2022 ]. Available from: <https://www.cdc.gov/vaccines/programs/iis/about.html>.

25. Michel JP, Gusmano M, Blank PR, Philp I. Vaccination and healthy ageing: How to make life-course vaccination a successful public health strategy. European Geriatric Medicine. 2010;1(3):155-65.

26. Fisher MP, Gurfinkel D, Szilagyi PG, Saville A, Albertin C, Breck A, et al. Supporting and sustaining centralized reminder/recall for immunizations: Qualitative insights from stakeholders. Vaccine. 2019;37(44):6601-8.

27. Jacobson Vann JC, Jacobson RM, Coyne-Beasley T, Asafu-Adjei JK, Szilagyi PG. Patient reminder and recall interventions to improve immunization rates. The Cochrane database of systematic reviews. 2018;1(1):Cd003941.

28. Tierney CD, Yusuf H, McMahon SR, Rusinak D, O'Brien MA, Massoudi MS, et al. Adoption of reminder and recall messages for immunizations by pediatricians and public health clinics. Pediatrics. 2003;112(5):1076-82.

29. Hurley LP, Beaty B, Lockhart S, Gurfinkel D, Breslin K, Dickinson M, et al. RCT of Centralized Vaccine Reminder/Recall for Adults. American journal of preventive medicine. 2018;55(2):231-9.

30. Ozawa S, Yemeke TT, Evans DR, Pallas SE, Wallace AS, Lee BY. Defining hard-to-reach populations for vaccination. Vaccine. 2019;37(37):5525-34.

31. Poudel A, Lau ETL, Deldot M, Campbell C, Waite NM, Nissen LM. Pharmacist role in vaccination: Evidence and challenges. Vaccine. 2019;37(40):5939-45.
